# Beyond blood-brain barrier disruption and molecular weight: compartmental kinetics of S100B and NSE for neurological prognostication after cardiac arrest

**DOI:** 10.1186/s13054-025-05572-8

**Published:** 2025-08-04

**Authors:** So-Young Jeon, Changshin Kang, Yenho You, Jung Soo Park, Jin Hgon Min, Wonjoon Jeong, Hyun Shik Ryu, Jiyoung Choi, Byung Kook Lee

**Affiliations:** 1 Department of Emergency Medicine, College of Medicine, Chungnam National University, 266 Munwha-ro, Jung-gu, Daejeon, 35015 Republic of Korea; 2 Department of Emergency Medicine, Chungnam National University Hospital, Daejoen, Republic of Korea; 3 Department of Emergency Medicine, Chonnam national University Medical School, Chonnam National University Hospital, Gwangju, Republic of Korea

**Keywords:** Biomarker, Blood-brain barrier integrity, Compartment kinetics, Cerebrospinal fluid, Molecular weight, Neuron-specific enolase, Out-of-hospital cardiac arrest, Prognostication timing, S100 calcium-binding protein

## Abstract

**Background:**

The prognostic value of serum biomarkers after out-of-hospital cardiac arrest (OHCA) depends on timing, but the physiological basis remains unclear. We investigated whether blood–brain barrier (BBB) integrity and biomarker-specific properties explain the time-dependent differences in prognostic performance.

**Methods:**

This retrospective study included comatose adult OHCA survivors who underwent paired serum and cerebrospinal fluid (CSF) measurements of neuron-specific enolase (NSE; 47 kDa) and S100 calcium-binding protein B (S100B; 21 kDa) at 0 (H0), 24 (H24), 48 (H48), and 72 (H72) h after return of spontaneous circulation. BBB disruption was assessed using the CSF/serum albumin quotient (Q_A_). Prognostic performance was assessed using AUC analysis for 6-month poor neurological outcome (Cerebral Performance Category 3–5).

**Results:**

Among 111 patients (59% poor outcome), 646 serum and 620 CSF samples were analyzed. BBB disruption was more severe in the poor outcome group at all timepoints (all *P* < 0.001), peaking at H24 (Q_A_ 0.0282 [IQR 0.0150–0.120]) and remaining elevated at H72 (0.0228 [IQR 0.0147–0.0598]). In the poor outcome group, serum S100B levels peaked at H0 (0.80 ng/mL [IQR 0.39–2.81]) and declined despite a persistent elevation in CSF levels at or above the upper detection limit (≥ 30 ng/mL). Conversely, NSE levels progressively increased in both compartments, with serum and CSF levels increasing in parallel over time. Serum NSE concentrations showed a time-dependent improvement in prognostic accuracy, peaking at H72 (AUC 0.88), whereas S100B concentrations maintained stable performance across all timepoints (AUCs 0.79–0.85, all *P* > 0.4). Notably, the prognostic performance of S100B remained relatively consistent regardless of BBB disruption severity, whereas NSE showed progressively improved predictive accuracy with increased BBB disruption. Across all timepoints, CSF biomarkers—particularly S100B and NSE—showed consistently higher AUCs than serum, suggesting superior prognostic utility.

**Conclusions:**

Serum NSE levels closely reflect the degree of BBB disruption and CSF levels, while S100B exhibits a transient early-phase profile, with decreased serum detectability over time, even in the presence of sustained CSF elevation or severe BBB disruption. These findings highlight the importance of interpreting biomarker kinetics across compartments and timepoints rather than relying on molecular weight or BBB status alone.

**Supplementary Information:**

The online version contains supplementary material available at 10.1186/s13054-025-05572-8.

## Background

Out-of-hospital cardiac arrest (OHCA) remains a leading cause of global mortality and long-term neurological disability [1–3]. Despite advances in post-resuscitation care, including targeted temperature management (TTM), accurately predicting neurological outcomes early after the return of spontaneous circulation (ROSC) remains a clinical challenge [4–6].

Among the available prognostic tools, serum biomarkers have the advantage of being quantitative and unaffected by sedatives and neuromuscular blockers commonly used in the post-cardiac arrest period [4]. Current international guidelines recommend the use of neuron-specific enolase (NSE), a biomarker of sustained neuronal injury, measured at 48–72 h after ROSC [4, 7]. S100 calcium-binding protein B (S100B) has also been extensively studied; most reports have found that serum concentrations measured within the first 24 h—particularly immediately post-ROSC or at 24 h—are independently associated with neurological outcomes [4, 7–10]. However, the physiological basis for these time-dependent patterns remains unclear. Prior studies have hypothesised that molecular weight and blood–brain barrier (BBB) permeability can explain these differences, with NSE (~ 78 kDa) being larger and S100B (~ 21 kDa) smaller [8, 11]. However, this framework may be overly simplistic, as biomarker-specific factors such as cellular origin, compartmental kinetics, clearance dynamics, and BBB transport mechanisms may also play a crucial role.

As only few studies have incorporated cerebrospinal fluid (CSF) biomarker measurements or directly assessed BBB integrity, our understanding of how serum levels reflect central nervous system (CNS) injury remains limited [12, 13]. To our knowledge, no study has simultaneously evaluated serum and CSF concentrations of NSE and S100B in OHCA survivors using a standardised marker of BBB disruption.

Therefore, in this study, we aimed to compare the serum and CSF levels of NSE and S100B in OHCA survivors stratified by BBB integrity (intact vs. disrupted, and by degree of disruption). We also assessed whether their prognostic performance reflects molecular size, BBB permeability, or compartment-specific dynamics. BBB disruption was quantified using the CSF-to-serum albumin quotient (Q_A_) [14, 15]. We hypothesised that the differences in biomarker behaviour would extend beyond molecular weight and BBB integrity and instead be shaped by biomarker-specific kinetics and clearance mechanisms.

## Methods

### Study design and population

This retrospective observational study analysed prospectively collected registry data from adult comatose OHCA survivors treated with TTM at Chungnam National University Hospital (CNUH) in Daejeon, Korea, between May 2018 and December 2023. A subset of this registry (67 patients from May 2018 to December 2019) overlapped with that in a previously published study on the association between BBB disruption and neurological outcomes [16]. The Institutional Review Board (IRB) of CNUH approved this study (CNUH-2024-06-045), and written informed consent was obtained from all patients and/or their legal guardian(s) and recorded in a database.

Adult patients (≥ 18 years) who survived OHCA, received TTM, and underwent lumbar catheter insertion for CSF albumin measurement were included. Exclusion criteria included OHCA due to trauma, ineligibility for lumbar catheter placement, absence of NSE and S100B testing within 72 h post-ROSC, pre-existing poor neurological status (e.g. coma or vegetative state), extracorporeal membrane oxygenation (ECMO) treatment, or lack of next of kin to provide consent for lumbar catheter placement. Lumbar catheter placement was considered ineligible if brain computed tomography scans revealed severe cerebral oedema (e.g. obliteration of the basal cisterns) or an occult intracranial mass lesion or if the patient was receiving antiplatelet or anticoagulation therapy or had coagulopathy (platelet count < 40 × 10³/µL or international normalised ratio > 1.5) [15].

### Data collection

We extracted the following data from a prospective registry: age, sex, Charlson Comorbidity Index (CCI) score, witnessed collapse, bystander cardiopulmonary resuscitation (CPR), first monitored rhythm (shockable vs. non-shockable), aetiology of cardiac arrest (cardiac vs. non-cardiac), no-flow time (collapse to CPR), low-flow time (CPR to ROSC), time from ROSC to lumbar catheter insertion, and NSE, S100B, and albumin levels in the serum and CSF at 0 (H0), 24 h (H24), 48 (H48), and 72 h (H72). “H0” refers to the first available post-ROSC serum or CSF sample, typically obtained at a median of 4.5 h after ROSC. This timing reflects the earliest feasible sampling window in our institutional protocol and is used as the “baseline” timepoint throughout the study. Missing data were not imputed; patients with unavailable measurements at a given timepoint were excluded from the respective receiver operating characteristic (ROC) analyses. Neurological outcomes were assessed 6 months after the ROSC using the Glasgow–Pittsburgh Cerebral Performance Category (CPC) scale and were dichotomised as good (CPC 1–2) or poor (CPC 3–5). Outcomes were determined through face-to-face or telephone interviews conducted by an emergency physician or a neurologist who was fully informed of the study protocol and blinded to the patient’s prognosis. A poor neurological outcome was designated as the primary endpoint.

### TTM protocol

Comatose OHCA survivors were managed according to our previously published TTM protocols [16], initiated as early as possible upon arrival at the emergency department or after ROSC. Target temperatures of 33–36 °C were maintained for 24 h using the Arctic Sun^®^ 5000 (BD, Franklin Lakes, NJ, USA), a feedback-controlled surface cooling device. After the maintenance phase, patients were gradually rewarmed to 37 °C at a controlled rate of 0.25 °C per h. Sedatives (midazolam: 0.05 mg/kg IV bolus followed by a titrated continuous infusion of 0.05–0.2 mg/kg/h) and neuromuscular blocking agents (cisatracurium: 0.15 mg/kg IV bolus followed by an infusion of up to 5 µg/kg/min) were administered throughout the TTM process. Seizure activity was monitored using amplitude-integrated electroencephalography (EEG) or bispectral index monitoring.

Prior to March 2022, our institution consistently applied a target temperature of 33 °C. After this date, the attending physician determined whether to apply 33–36 °C based on the patient’s haemodynamic stability and aetiology of the cardiac arrest (cardiac vs. non-cardiac). All patients received comprehensive post-cardiac arrest care following our standardised institutional protocol, which aligns with international guidelines. Notably, while all patients in this study underwent TTM, many hospitals have since transitioned to broader temperature control strategies.

Neurological prognostication was performed after rewarming and at least 72 h post-ROSC, using a multimodal approach in accordance with international post–cardiac arrest care guidelines [4]. This included clinical examination, electrophysiological monitoring (EEG and/or amplitude-integrated EEG), serial serum NSE measurements, and neuroimaging (CT and MRI). In South Korea, withdrawal of life-sustaining therapy (WLST) was legally prohibited prior to February 2018 unless brain death was confirmed. Even after legalization, WLST remains uncommon in clinical practice due to prevailing cultural, ethical, and societal norms [17–19]. At our institution, WLST is generally not performed during TTM unless brain death is confirmed and organ donation is being considered. In this study, no patients underwent WLST during TTM; all received full intensive care. Nonetheless, some were declared dead based on circulatory or neurological criteria despite ongoing life-sustaining support.

### Measurement of BBB integrity

As part of our institution’s post-resuscitation care protocol, a radial artery catheter and a Hermetic™ (Integra Neurosciences, Plainsboro, NJ, USA) lumbar catheter were inserted between the third and fourth lumbar vertebrae, provided that informed consent had been obtained from the patient’s next of kin. The distal end of the lumbar catheter was connected to a LiquoGuard system (Möller Medical GmbH & Co KG, Fulda, Germany) to enable CSF drainage.

At our institution, lumbar catheter placement was incorporated into a standardised post-resuscitation care protocol for comatose survivors of OHCA undergoing TTM. The initial implementation of this protocol was approved by the IRB of CNUH and included prospective observational studies [15, 20–22]. Subsequently, lumbar drainage was continued as part of routine clinical practice at our institution, with informed consent obtained from each patient’s legal surrogate. The procedure was performed only after confirming patient eligibility and securing surrogate consent. Lumbar drains were removed 72 h after ROSC to minimise the risk of infection, and no additional prophylactic antibiotics were administered. No procedure-related complications—such as infection, bleeding, or herniation—were observed throughout the study period [15, 23].

Blood and CSF samples for albumin analysis were collected immediately after ROSC (H0) and subsequently at 24-h intervals over a 72-h period (H24, H48, and H72). Using standard colorimetric and immunoassay methods (ARUP Laboratories, Salt Lake City, UT, USA), the values were measured as part of a comprehensive metabolic panel. BBB integrity was assessed using Q_A_, which is currently the most accessible and reliable biomarker for evaluating BBB permeability. Q_A_ was calculated as the ratio of albumin concentrations in the CSF and serum. BBB disruption has been previously classified as follows: intact (Q_A_ < 0.007) or disrupted, which is further categorised as mild (Q_A_: 0.007–0.01), moderate (Q_A_: 0.01–0.02), or severe (Q_A_: >0.02) [15, 16, 20].

### Quantification of serum and CSF concentrations of NSE and S100B

Levels of blood and CSF biomarkers, including NSE and S100B, were collected at 24-h intervals for up to 72 h post-ROSC via a radial arterial catheter and analysed at Green Cross Laboratory (GC Labs, Yongin, Gyeonggi-do). NSE and S100B levels were determined using an electrochemiluminescence immunoassay with Elecsys NSE^®^ (COBAS e801; Roche Diagnostics, Rotkreuz, Switzerland) and Elecsys S100^®^ (COBAS e411; Roche Diagnostics, Mannheim, Germany), respectively. The measurement ranges were 0.1–300 ng/mL for NSE and 0.005–30 ng/mL for S100B. In accordance with the laboratory’s standard protocol, serum aliquots grossly contaminated with blood or exhibiting excessive hemolysis—based on a predefined hemolysis index threshold—were automatically excluded prior to analysis [21]. All assays were performed in compliance with national and international quality standards. However, detailed hemolysis indices and the number of excluded samples were not recorded in the institutional registry.

### Statistical analysis

Categorical variables are presented as frequencies with percentages, and continuous variables are presented as medians with interquartile ranges (IQR 25–75) because of their non-normal distribution, as determined with the Shapiro–Wilk test. Non-parametric tests were used to account for the skewed distribution of many parameters. Categorical variables were compared using the chi-square or Fisher’s exact test, while continuous variables were analysed using the Mann–Whitney *U* test. ROC curves with 95% confidence intervals (CIs) were used to assess the predictive performance of NSE and S100B levels for poor neurological outcomes at 6 months based on measurements at sequential time points—H0, H24, H48, and H72. The area under the ROC curve (AUC) was calculated for each time point and compared using the DeLong test [24]. We selected biomarker cut-off values at each time point that achieved 100% specificity (i.e., false positive rate [FPR] = 0), thereby avoiding any false positives among patients with good neurological outcomes. This stringent threshold approach (as opposed to using a criterion like maximizing Youden’s index) was chosen to prioritize certainty in predicting poor outcomes, at the expense of sensitivity. For each time point and biomarker, we report the AUC and the sensitivity, specificity, positive predictive value, and negative predictive value at the chosen cut-off, all with 95% CIs. Prognostic performance was classified based on AUC values as follows: poor (0.50–0.69), fair (0.70–0.79), good (0.80–0.89), and excellent (0.90–1.00) [25]. A two-tailed *P*-value of < 0.05 was considered significant. Statistical analyses were conducted using SPSS version 24.0 (IBM Corp., Armonk, NY, USA) and MedCalc^®^ Statistical Software version 20.118 (MedCalc Software Ltd, Ostend, Belgium).

## Results

### Patient characteristics

**Fig. 1:**
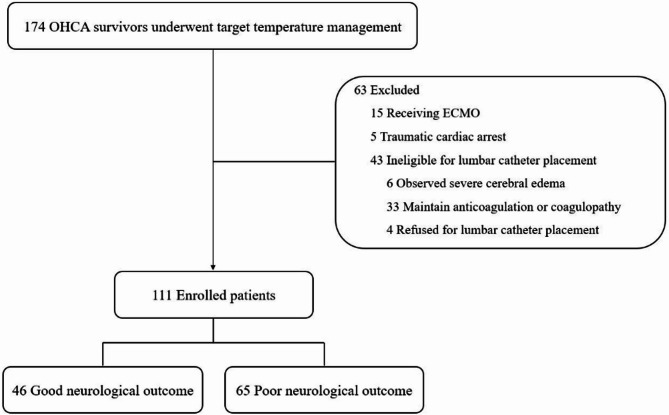
Flow diagram of the patients included in this study. OHCA, out-of-hospital cardiac arrest; ECMO, extracorporeal membrane oxygenation

Among 174 comatose OHCA survivors treated with TTM during the study period, 111 patients were included in our study after excluding those who did not undergo lumbar drainage catheter insertion after resuscitation (*n* = 43), those who underwent ECMO (*n* = 15), and those with traumatic cardiac arrest (*n* = 5). At 6 months post-ROSC, 65 of 111 patients (59%) had poor neurological outcomes—the defined target condition in this study—while 46 (41%) had good outcomes (Fig. 1). The demographic and clinical characteristics of the patients, stratified according to neurological outcome at 6 months, are summarised in Table 1. Compared to the good outcome group, the poor outcome group had lower rates of witnessed events, bystander CPR, shockable rhythm, and cardiac aetiology, as well as longer no- and low-flow times, lower GWR values, and higher rCAST scores. However, there were no significant differences between the groups in the time interval from ROSC to computed tomography scan or lumbar puncture (LP), nor in age, sex, and CCI (Table 1).

**Table 1:** Baseline demographic data and clinical characteristics

Characteristics		Cohort (*n* =111)	Good neurological outcome (*n* =46)	Poor neurological outcome (*n* =65)	*P*-value^a^
Age, years	57.0 (42.0–69.0)		55.0 (37.8–66.5)	57.0 (43.5–70.0)	0.21
Male sex	81 (73.0)		37 (80.4)	44 (67.7)	0.19
Charlson Comorbidity Index score	2.0 (0.0–4.0)		1.0 (0.0–3.0)	1.5 (0.0–3.0)	0.75
Arrest characteristics					
Witnessed arrest	64 (57.7)		37 (80.4)	27 (41.5)	< 0.001
Bystander CPR	78 (70.3)		38 (82.6)	40 (61.5)	0.02
Shockable rhythm	33 (29.7)		27 (58.7)	6 (9.2)	< 0.001
Cardiac aetiology	42 (37.8)		28 (60.9)	14 (21.5)	< 0.001
No flow time, min	2.0 (0–13.0)		0.0 (0.0–1.0)	5.0 (0.0–23.5)	< 0.001
Low flow time, min	19.0 (10.0–30.0)		15.0 (8.0–18.5)	29.5 (20.5–43.0)	< 0.001
rCAST score,	11.3 (8.5–14.5)		8.5 (4.3–11.0)	13.0 (10.0–16.0)	< 0.001
ROSC to HCT time, min	61 (39–94)		57 (37–80)	72 (41–109)	0.14
GWR value	1.24 (1.17–1.3)		1.27 (1.22–1.31)	1.21 (1.16–1.27)	0.002
Lumbar puncture time, hr	4.5 (3.4–5.9)		4.1 (3.0–5.8)	4.7 (4.0–6.0)	0.06

Continuous variables are presented as median (interquartile range); categorical variables as number (%)

^a^ P values are based on *χ*2 test for categorical variables and the Mann-Whitney U test for continuous variables

CCI, Charlson comorbidity index; CPR, cardiopulmonary resuscitation; rCAST, revised post-cardiac arrest syndrome; ROSC, return of spontaneous circulation; HCT, head computed tomography; GWR, grey-to-white matter ratio

Time to lumbar puncture was calculated from the time of ROSC

### BBB disruption and Temporal changes in CSF and serum biomarker concentrations

In the overall cohort, BBB disruption, as measured with the Q_A_, was significantly higher in patients with poor neurological outcomes at all timepoints (all *P* < 0.05) (see Additional file 1, Table S1). As shown in Additional file 1 (Figure S1), patients with moderate (67%) or severe BBB disruption (83%) exhibited a higher proportion of poor neurological outcomes than those with an intact barrier (27%) or mild disruption (55%) (*P* < 0.001). Furthermore, severe BBB disruption was most frequently observed at H24. The disruption peaked at H24 (median Q_A_ 0.0282) and remained elevated at H72 (median Q_A_ 0.0228).

**Fig. 2:**
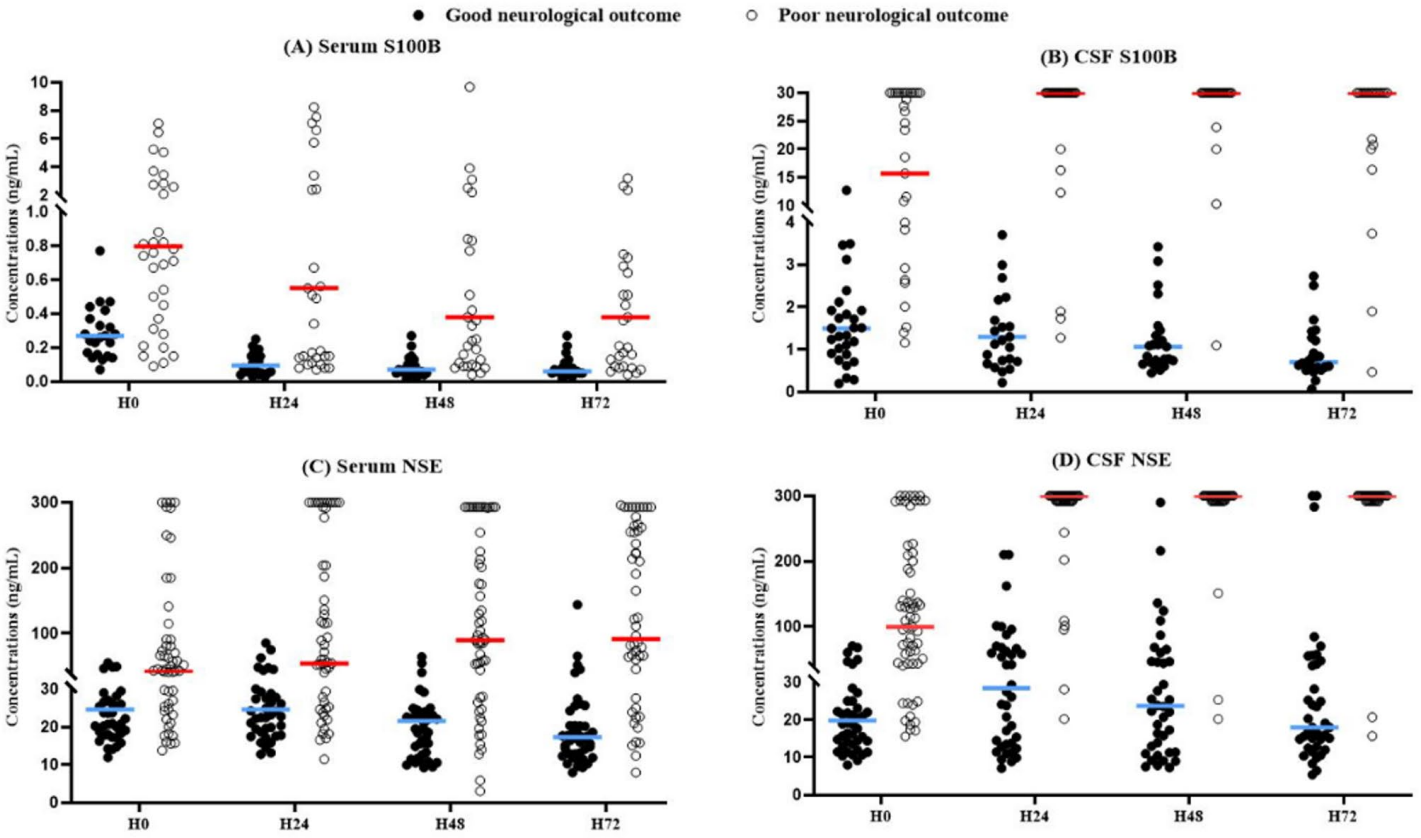
Temporal changes in serum and cerebrospinal fluid concentrations of S100B and NSE according to neurological outcomes. S100B and NSE concentrations were measured at four time points: baseline (H0) and at 24, 48, and 72 h post-ROSC (H24, H48, and H72). Each panel compares biomarker levels between patients with good and poor neurological outcomes: (**A**) Serum S100B, (**B**) CSF S100B, (**C**) Serum NSE, and (**D**) CSF NSE. ● indicates good neurological outcomes; ○ indicates poor neurological outcomes. Horizontal bars represent median values. H0 represents the baseline sample collected at a median of 4.5 h after ROSC. NSE, neuron-specific enolase; S100B, S100 calcium-binding protein B; CSF, cerebrospinal fluid; ROSC, return of spontaneous circulation

Figure 2 and Additional file 1 (Table S2) demonstrate that, in patients with poor neurological outcome, serum S100B concentrations peaked at H0 (median 0.80 ng/mL) and rapidly declined over time, reaching a median of 0.38 ng/mL at H72, despite consistently elevated CSF concentrations (median ≥ 30 ng/mL from H24 to H72). In contrast, NSE concentrations progressively increased in both the serum and CSF compartments, with the highest levels observed at H72 (median 91.3 ng/mL in the serum and 300 ng/mL in the CSF), maintaining a parallel temporal pattern

Table 2 shows stratified biomarker concentrations according to the degree of BBB disruption and neurological outcome. In the serum, NSE concentrations showed higher median values with greater BBB disruption, increasing from 24.1 ng/mL in the intact group to 71.7 ng/mL in the severe group, with consistently higher levels observed in the poor outcome group than in the good outcome group. In contrast, serum S100B concentrations did not follow the same compartmental pattern as did NSE. Despite a progressive increase in CSF S100B concentrations across BBB disruption categories—from 1.51 ng/mL in the intact group to 30.0 ng/mL in the severe group (upper detection limit)—serum levels increased only modestly and inconsistently (0.14 to 0.67 ng/mL), with no clear relationship between CSF and serum concentrations.

**Table 2:** Comparison of serum and CSF concentrations of NSE and S100B according to neurological outcome across degrees of BBB disruption

Degree of BBB disruption	Overall cohort	Good neurological outcome	Poor neurological outcome	*P*-value^a^
**Serum**				
**Neuron-specific enolase**,** ng/ml**				
Intact	24.1 (17.1–34.4), 114^b^	22.5 (15.8–31.1), 83^b^	29.8 (21.0–45.6), 31^b^	0.007
Mild	26.1 (18.6–40.1), 64^b^	20.3 (16.3–29.5), 35^b^	37.8 (22.5–56.9), 29^b^	<0.001
Moderate	28.0 (20.1–83.8), 77^b^	20.3 (17.3–24.9), 25^b^	41.4 (24.9–125.3), 52^b^	<0.001
Severe	71.7 (27.9–254.0), 127^b^	22.2 (15.2–27.3), 22^b^	97.2 (43.5–292.9), 105^b^	<0.001
**S100 calcium-binding protein B**,** ng/ml**				
Intact	0.14 (0.07–0.28), 52^b^	0.09 (0.06–0.18), 36^b^	0.37 (0.17–1.64), 16^b^	<0.001
Mild	0.24 (0.07–0.51), 30^b^	0.10 (0.05–0.21), 16^b^	0.52 (0.25–0.79), 14^b^	<0.001
Moderate	0.15 (0.08–0.51), 49^b^	0.12 (0.08–0.30), 17^b^	0.20 (0.08–0.70), 32^b^	0.21
Severe	0.67 (0.15–2.39), 72^b^	0.17 (0.05–0.21), 11^b^	0.82 (0.16–2.57), 61^b^	0.001
**Cerebrospinal fluid**				
**Neuron-specific enolase**,** ng/ml**				
Intact	25.1 (14.2–70.1), 112^b^	20.8 (11.5–36.0), 82^b^	132.0 (38.8–292.1), 30^b^	<0.001
Mild	43.3 (17.9–124.5), 65^b^	19.9 (14.9–45.7), 36^b^	140.0 (43.9–296.5), 29^b^	<0.001
Moderate	285.0 (25.0–300.0), 75^b^	20.2 (11.6–34.1), 24^b^	292.9 (151.0–300.0), 51^b^	<0.001
Severe	300.0 (291.4–300.0), 126^b^	34.8 (17.3–72.8), 22^b^	300.0 (292.9–300.0), 104^b^	<0.001
**S100 calcium-binding protein B**,** ng/ml**				
Intact	1.51 (0.71–4.29), 66^b^	1.12 (0.63–1.56), 47^b^	7.17 (4.16–26.7), 19^b^	<0.001
Mild	1.53 (0.78–7.31), 37^b^	0.84 (0.65–1.67), 21^b^	7.47 (2.71–30.00), 16^b^	<0.001
Moderate	8.98 (1.33–30.00), 54^b^	1.24 (0.67–2.71), 21^b^	23.90 (9.29–30.00), 33^b^	<0.001
Severe	30.00 (12.32–30.00), 75^b^	1.30 (1.00–2.41), 13^b^	30.00 (30.00–30.00), 62^b^	<0.001

Data are presented as median (interquartile range)

^a^P values are based on the Mann-Whitney U test for continuous variables

^b^Number of samples included in each analysis. The total number of patients: 111 (46 with good neurological outcome and 65 with poor neurological outcome)

NSE, neuron-specific enolase; S100B, S100 calcium-binding protein B; CSF, cerebrospinal fluid

### Prognostic accuracy of S100B and NSE across timepoints and BBB integrity

Table 3 shows the prognostic performance of each biomarker according to the severity of BBB disruption. For NSE, the AUC increased from 0.66 in the intact group to 0.91 in the severe group (*P* < 0.001), with sensitivity rising from 9.7 to 62.9%, while specificity remained at 100%. The highest predictive performance was observed in the severe disruption group (AUC 0.91). In contrast, S100B achieved its highest AUC (0.92) in the mild BBB disruption group, with a sensitivity of 71.4% at 100% specificity. However, the AUCs remained relatively stable in the intact (0.84) and severe (0.80) groups, and sensitivity markedly dropped in the moderate group (15.6%), despite preserved specificity.

**Table 3:** Diagnostic performance of serum biomarkers across grades of blood–brain barrier disruption for poor neurological outcome

BBB disruption grade	AUC (95%CI)	Cut-off value	Sensitivity (95% CI)	Specificity (95% CI)	PPV (95% CI)	NPV (95% CI)	*P*-value^b^
**Neuron-specific enolase**							
**Degree of BBB disruption**							
Intact, 114^a^	0.66 (0.55–0.78)	>85.3	9.7 (2.0–25.8)	100.0 (95.7–100.0)	100	74.8 (72.5–76.9)	<0.001
Mild, 64^a^	0.78 (0.66–0.89)	>51.6	27.6 (12.7–47.2)	100.0 (90.0–100.0)	100	62.5 (57.1–67.6)	0.04
Moderate, 77^a^	0.81 (0.72–0.91)	>54.6	48.1 (34.0–62.4)	100.0 (86.3–100.0)	100	48.1 (41.6–54.6)	0.07
Severe, 127^a^	0.91 (0.86–0.96)	>65.3	62.9 (52.9–72.1)	100.0 (84.6–100.0)	100	36.1 (30.5–42.0)	Reference
**S100 calcium binding protein B**							
**Degree of BBB disruption**							
Intact, 52 ^a^	0.84 (0.71–0.97)	>0.42	43.8 (19.8–70.1)	100.0 (90.3–100.0)	100	80.0 (72.2–86.0)	0.32
Mild, 30 ^a^	0.92 (0.83–1.00)	>0.33	71.4 (41.9–91.6)	100.0 (79.4–100.0)	100	80.0 (63.6–90.2)	Reference
Moderate, 49 ^a^	0.61 (0.44–0.77)	>1.6	15.6 (5.3–32.8)	100.0 (80.5–100.0)	100	38.6 (35.2–42.2)	0.011
Severe, 72 ^a^	0.80 (0.68–0.92)	>0.77	50.8 (37.7–63.9)	100.0 (71.5–100.0)	100	26.8 (22.1–32.1)	0.13
NSE overall, 382 ^a^	0.83 (0.79–0.86)	>144	30.0 (24.4–36.4)	100.0 (98.0–100.0)	100	52.0 (49.9–54.1)	N/A
S100B overall, 203 ^a^	0.83 (0.77–0.87)	>1.6	30.2 (22.6–38.6)	100.0 (95.9–100.0)	100	48.4 (45.6–51.1)	N/A

All cut-off values were determined based on 100% specificity

^a^Number of samples included in the analysis

^b^P values are based on the DeLong test for comparison of the area under the receiver operating characteristic curve (reference: BBB disruption state and time with the lowest AUC value)

BBB, blood-brain barrier; AUC, area under the receiver operating characteristic curve; CI, confidence interval; PPV, positive predictive value; NPV, negative predictive value

N/A = not applicable; P values are not calculated for overall comparisons across BBB grades

To explore the reduced sensitivity of S100B in the moderate BBB disruption group, we analyzed the causes of poor neurological outcomes (Additional file 1, Table S3). Among the nine patients with CPC 3–5 in this group, only one patient died from neurological causes, while three died from extracerebral complications. In contrast, of the 39 patients with poor outcomes in the severe disruption group, 20 died due to neurological injury.

Across timepoints, the AUC for serum NSE progressively increased over time, from 0.73 at H0 to 0.88 at H72 (*P* = 0.001 vs. H0), whereas S100B maintained relatively stable performance from 0.79 to 0.85 (all *P* > 0.4), showing no significant time-dependent changes (Additional file 1, Figure S2).

**Fig. 3:**
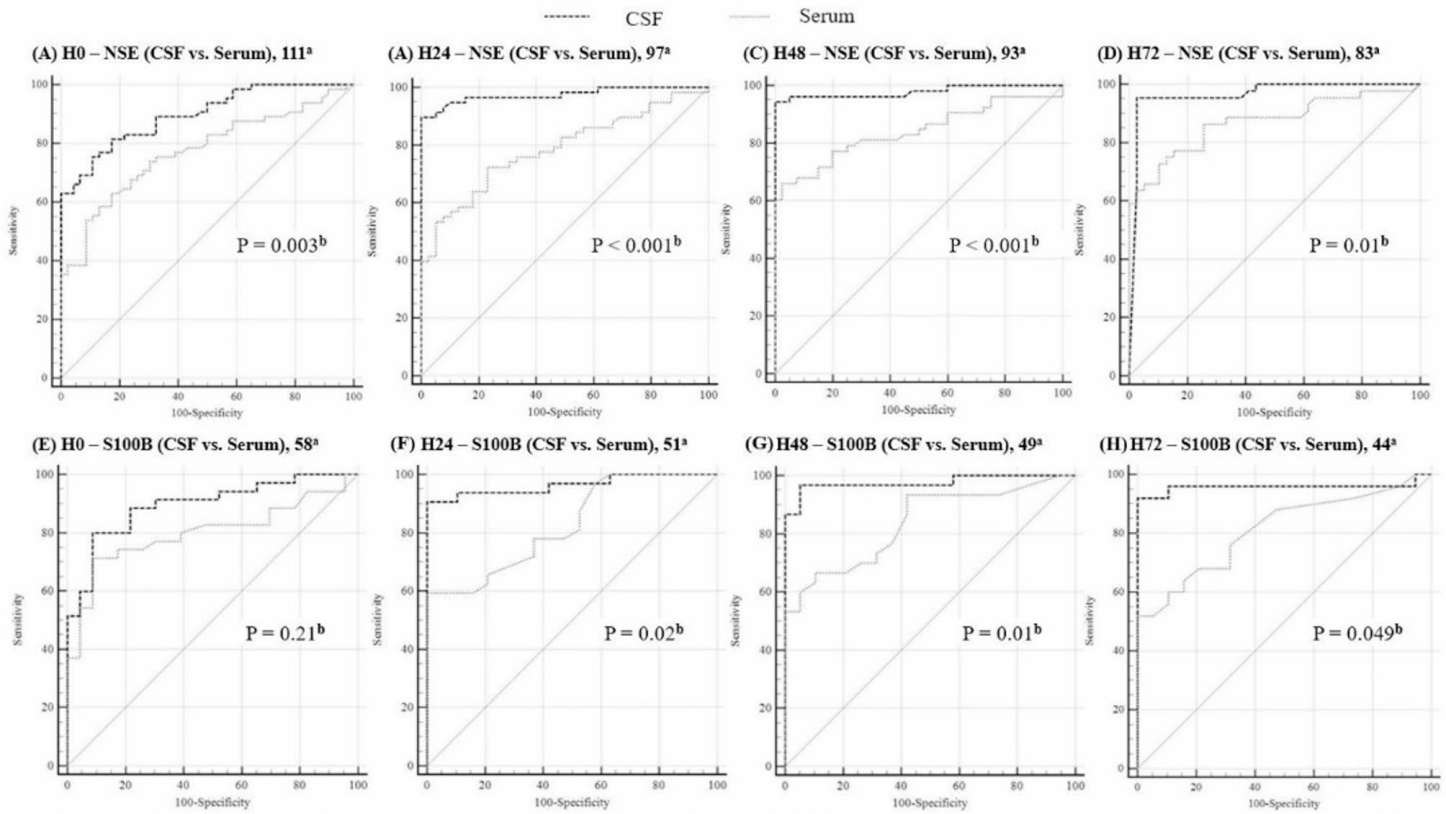
Comparison of ROC curves for predicting poor neurological outcome (CPC 3–5) at 6 months after OHCA using CSF and serum concentrations of NSE and S100B across four timepoints (H0, H24, H48, H72). Panels (**A**–**D**) show ROC curves for NSE; panels (**E**–**H**) show ROC curves for S100B. CSF and serum performance are compared at each timepoint. ^a^Number of samples included in the analysis; ^b^P-values are based on the DeLong test for AUC comparisons. H0 represents the baseline sample collected at a median of 4.5 h after ROSC. AUC, area under the curve; ROC, receiver operating characteristic; CPC, Cerebral Performance Category; NSE, neuron-specific enolase; S100B, S100 calcium-binding protein B; CSF, cerebrospinal fluid; OHCA, out-of-hospital cardiac arrest

To further characterize the prognostic utility of S100B and NSE, we compared their predictive performance in CSF and serum across four timepoints (H0, H24, H48, and H72). As shown in Fig. 3, CSF biomarkers consistently demonstrated higher AUC values than their serum counterparts, with the greatest differences observed at H24 and H48 (*P* < 0.05 for all comparisons).

Contingency analyses based on cut-off values corresponding to 100% specificity (Table 4) further demonstrated that CSF NSE exhibited greater sensitivity than serum NSE from H24 onward. Likewise, CSF S100B outperformed its serum counterpart, particularly at later timepoints.

**Table 4:** Prognostic performance of serum and CSF NSE and S100B at different time points for predicting poor neurological outcome, with cut-off values based on 100% specificity

Biomarker	Time point (Number analyzed)	Cut-off (ng/mL)	AUC (95% CI)	Sensitivity (95% CI)	Specificity (95% CI)	PPV	NPV (95% CI)	TP	FP	TN	FN
Serum NSE	H0 (*n*=111)	>54.8	0.77 (0.68–0.85)	35 (24–48)	100 (92–100)	100	52 (48–57)	23	46	0	42
	H24 (*n*=105)	>85.3	0.79 (0.70–0.86)	41 (29–54)	100 (92–100)	100	55 (50–60)	25	44	0	36
	H48 (*n*=105)	>64.2	0.85 (0.77–0.91)	59 (46–72)	100 (92–100)	100	65 (59–72)	35	46	0	24
	H72 (*n*=99)	>144	0.88(0.80–0.94)	43 (29–57)	100 (92–100)	100	59 (54–65)	23	45	0	31
CSF NSE	H0 (*n*=111)	>70.2	0.90 (0.83–0.95)	63 (50–75)	100 (92–100)	100	66 (58–73)	41	46	0	24
	H24 (*n*=98)	>210	0.97 (0.92–0.996)	90 (79–96)	100 (91–100)	100	87 (76–93)	52	40	0	6
	H48 (*n*=93)	>290	0.98 (0.93–0.998)	94 (84–99)	100 (92–100)	100	93 (82–98)	50	40	0	3
	H72 (*n*=83)	>300^a^	N/A^b^	0 (0–0)	100 (100–100)	N/A^b^	47 (37–58)	0	0	39	44
Serum S100B	H0 (*n*=59)	>1.6	0.80 (0.68–0.90)	36 (21–54)	100 (85–100)	100	50 (44–56)	13	23	0	23
	H24 (*n*=57)	>0.25	0.84 (0.71–0.92)	60 (42–76)	100 (85–100)	100	61 (51–70)	21	22	0	14
	H48 (*n*=57)	>0.27	0.86 (0.75–0.94)	57 (39–73)	100 (85–100)	100	60 (50–68)	20	22	0	15
	H72 (*n*=53)	>0.27	0.85 (0.73–0.93)	55 (36–73)	100 (85–100)	100	61 (52–70)	17	22	0	14
CSF S100B	H0 (*n*=68)	>12.72	0.92 (0.83–0.97)	51 (35–65)	100 (88–100)	100	60 (53–68)	20	29	0	19
	H24 (*n*=60)	>6.83	0.97 (0.88–0.995)	89 (73–97)	100 (86–100)	100	86 (71–94)	31	25	0	4
	H48 (*n*=57)	>7.97	0.98 (0.91–1.000)	88 (71–97)	100 (86–100)	100	86 (71–94)	28	25	0	4
	H72 (*n*=50)	>2.73	0.96 (0.87–0.996)	92 (75–99)	100 (86–100)	100	92 (76–98)	24	24	0	2

Cut-off values selected to ensure 100% specificity at each timepoint

^a^ Upper limit of assay detection

^b^ N/A: Not applicable or not calculable due to lack of positive predictions

H72 CSF NSE values may have been saturated at the assay’s upper limit (300.0 ng/mL), preventing estimation of AUROC and PPV. “H0” represents the baseline sample collected at a median of 4.5 h after ROSC

CSF, cerebrospinal fluid; NSE, neuron-specific enolase; S100B, S100 calcium-binding protein B; AUC, area under the receiver operating characteristic curve; PPV, positive predictive value; NPV, negative predictive value; TP, true positive; FP, false positive; TN, true negative; FN, false negative; CI, confidence interval

### Comparison of prognostic performance of NSE and S100B based on BBB integrity

**Fig. 4:**
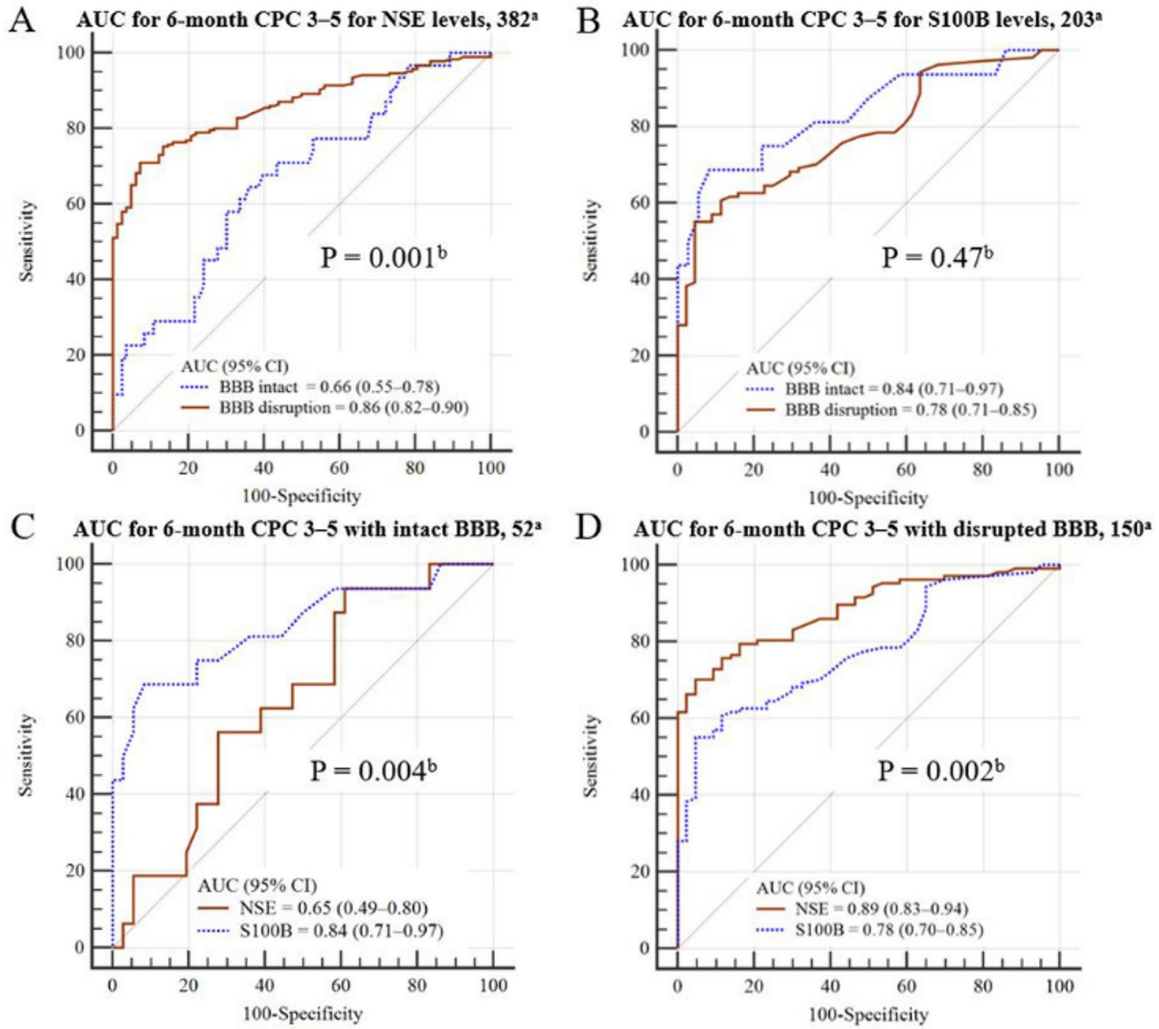
Comparative prognostic performance of NSE and S100B under intact and disrupted BBB conditions. (**A**) Comparison of ROC curves for NSE under intact and disrupted BBB conditions. (**B**) Comparison of ROC curves for S100B under intact and disrupted BBB conditions. (**C**) ROC curves comparing S100B and NSE in cases of an intact BBB. (**D**) ROC curves comparing NSE and S100B in cases of a disrupted BBB. a Number of samples included in the analysis; b P-values are based on the DeLong test for AUC comparisons. ROC, receiver operating characteristic; AUC, area under the receiver operating characteristic curve; NSE, neuron-specific enolase; BBB, blood-brain barrier; S100B, S100 calcium-binding protein B; CI, confidence interval

Figure 4 compares the prognostic performance of NSE and S100B under intact and disrupted BBB conditions. AUC analysis showed that NSE demonstrated better prognostic performance in cases with disrupted BBB than in those with intact BBB (AUC 0.86 vs. 0.66, *P* = 0.001; Fig. 4a). Conversely, S100B maintained a fair to good prognostic performance without significant differences between disrupted and intact BBB conditions (AUC 0.78 vs. 0.84, *P* = 0.47; Fig. 4b). In addition, in cases with intact BBB, S100B demonstrated a better prognostic performance than NSE (AUC 0.84 vs. 0.65, *P* = 0.004; Fig. 4c). When the BBB was disrupted, NSE outperformed S100B (AUC 0.89 vs. 0.78, *P* = 0.002), whereas the opposite trend was observed under intact conditions (Fig. 4d).

## Discussion

In this retrospective study, we observed distinct temporal and BBB-dependent prognostic patterns between NSE and S100B in OHCA survivors. NSE concentrations progressively increased in both the CSF and serum compartments over time, with parallel kinetics. Prognostic accuracy also improved with increasing BBB disruption, rising from an AUC of 0.66 in the intact group to 0.91 in the severe group and from 0.73 at H0 to 0.88 at H72. In contrast, S100B concentrations peaked early in the serum at H0 and declined thereafter despite persistently elevated CSF levels. Prognostic performance for S100B remained relatively stable across timepoints and BBB disruption categories (AUC 0.84 in intact vs. 0.80 in severe, *P* = 0.47), with the highest accuracy observed in cases of mild BBB disruption (AUC 0.92). Under intact BBB conditions, S100B outperformed NSE (AUC 0.84 vs. 0.65, *P* = 0.004), whereas the opposite was true when the BBB was disrupted.

Previous studies have reported that the degree of BBB disruption is correlated with the severity of hypoxic-ischemic brain injury (HIBI), suggesting that severe BBB disruption is associated with poor neurological outcomes [16, 20, 26]. In a recent prospective study by Yoo et al., involving 32 patients, the Q_A_ was measured every 2 h over a 24-h period following the ROSC [15]. The authors found that Q_A_ values in the Pittsburgh Cardiac Arrest Category (PCAC) 2 group remained below 0.01 at all time points, indicating normal or mild BBB disruption conditions. In contrast, Q_A_ values in the PCAC 3 and 4 groups exhibited marked increase in BBB permeability at approximately 14 and 22 h and 12 and 14 h post-ROSC, respectively. Consistent with these findings, our study demonstrated that compared with intact or mildly disrupted BBB function, moderate and severe BBB disruption was associated with poor neurological outcomes.

The temporal behavior of S100B has been characterized in prior studies, revealing two distinct patterns following brain injury. One group of studies supports the prognostic utility of S100B within the first 24 h post-ROSC [4, 7–10]. Deye et al. reported that increased S100B levels measured immediately after ROSC were associated with poor neurological outcomes and demonstrated the highest AUC values at that early timepoint. Similar findings were reported by Rosén et al. and Jang et al., who identified S100B as an early-phase marker of cerebral injury [27, 28]. These studies interpreted early S100B release as a consequence of astrocytic activation, low molecular weight (~ 21 kDa), and rapid transit across a mildly disrupted BBB. However, other studies—including the subgroup analyses from the TTM 24 vs. 48 h trial by Duez et al. and the TTM 33 vs. 36 °C study by Stammet et al.—have demonstrated that although serum S100B levels declined over time, prognostic performance either remained stable or improved at later timepoints [10, 29]. A recent report by Hu et al. also showed that S100B AUCs peaked after 24 h in certain subgroups. However, these studies did not provide detailed mechanistic explanations for this observation [30].

Our study showed similar patterns. While serum S100B levels peaked at H0, the highest prognostic accuracy was observed at H48 (AUC 0.86), and most notably in the mild BBB disruption group (AUC 0.92). In contrast, NSE exhibited a parallel increase in serum and CSF levels, with the strongest prognostic performance at H72 (AUC 0.88) and in the severe BBB disruption group (AUC 0.91). These findings highlight an important clinical feature. The relationship between time and prognostic accuracy for S100B may be more variable than previously assumed, even when considering BBB integrity and molecular weight. Furthermore, our subgroup analysis revealed that in the moderate BBB disruption group, several poor outcome cases were attributable to extracerebral causes. In contrast, neurological injury predominated in the severe group, suggesting that S100B may more accurately reflect brain-specific pathology when BBB disruption is more advanced. Taken together, these findings suggest that S100B differs from NSE not only in terms of compartmental kinetics but also in its underlying pathophysiological mechanisms. In addition to NSE and S100B, several emerging neurobiomarkers—such as neurofilament light chain (NfL), Tau, and glial fibrillary acidic protein (GFAP)—have shown promise for outcome prediction after cardiac arrest [31–33]. Among these, NfL (68 kDa), an axonal injury marker with a long half-life, has demonstrated superior predictive accuracy at 48–72 h post-ROSC (AUCs up to 0.95) in some studies, although its early-phase performance remains limited (AUC 0.66 immediately post-ROSC) [32, 33]. While direct data on BBB disruption and NfL kinetics in cardiac arrest are scarce, findings from other neurological disorders suggest that serum NfL largely reflects axonal damage rather than passive leakage [34]. Nonetheless, increased albumin quotients have been associated with elevated NfL levels in CSF and serum under conditions of severe BBB dysfunction [35]. GFAP (50 kDa)—an astrocytic marker akin to S100B—exhibits similar kinetics but reaches peak accuracy at later time points (AUC 0.88 at 72 h) [33]. Collectively, these observations support the notion that biomarker-specific kinetics, including cellular origin, half-life, and compartmental clearance, more strongly shape prognostic accuracy than molecular size or barrier permeability alone.

The added prognostic utility of CSF biomarkers—particularly NSE and S100B—was evident in our ROC-based comparisons, which showed significantly higher AUCs in CSF than in serum across multiple timepoints. This advantage was most pronounced between 24 and 48 h post-ROSC, suggesting that CSF-based analysis may improve prognostic precision in subgroups where serum biomarkers yield inconclusive results. Notably, at 72 h, although CSF NSE demonstrated high overall prognostic performance, its false-positive rate at the 100% specificity threshold could not be reliably estimated. This limitation arose because two patients with good neurological outcomes exhibited CSF NSE concentrations at the assay’s upper detection limit (300 ng/mL), precluding accurate distinction between true and false positives. Upon review, no distinctive clinical features (e.g., age, arrest rhythm, or comorbidities) were identified to explain this discrepancy. However, both patients had CSF S100B levels within the expected range for good outcomes (1.70 and 2.51 ng/mL, respectively). These findings highlight the need for further investigation into potential assay ceiling effects and the interpretation of high CSF NSE values when other biomarkers remain within normal prognostic ranges.

S100B is actively secreted into the CSF by astrocytes under acute stress conditions such as ischaemia, oxidative injury, and glutamate excitotoxicity [36, 37]. Unlike NSE, which is a cytoplasmic protein primarily released by necrotic neurons, S100B can be exocytosed from viable astrocytes shortly after injury. Owing to its small molecular size (~ 21 kDa), S100B can traverse a mildly disrupted or even intact BBB via passive diffusion or regulated transcytosis [38, 39]. Moreover, the BBB in the early post-ROSC phase is known to be functionally permeable despite having a preserved gross structure, allowing low-molecular-weight proteins such as S100B to reach the bloodstream [40]. These mechanisms likely explain the early serum spike of S100B observed in this and previous studies.

However, in the context of severe BBB disruption, we observed that serum S100B levels declined over time despite persistently elevated CSF levels that consistently reached the upper detection limit (≥ 30 ng/mL) of the assay. This dissociation highlights a key difference in compartmental behaviour among the biomarkers. S100B concentrations peaked early in the serum and declined thereafter, failing to mirror sustained elevation in CSF levels. In contrast, NSE concentrations increased in parallel across both compartments, showing synchronised kinetics and stronger alignment with the severity of BBB disruption. This dissociation between compartments can be explained by several interrelated mechanisms. First, in the presence of profound BBB damage, the structural and functional integrity of the glymphatic system and perivascular drainage pathways may be severely compromised [39]. Under such conditions, the normal efflux of proteins from the CSF to the bloodstream is impaired [36, 40]. Consequently, S100B that is actively secreted by astrocytes accumulates within the CSF compartment and becomes ‘trapped’, although a strong concentration gradient still exists. This phenomenon—termed compartmental trapping—has been described in ischaemia and traumatic brain injury models, wherein solutes fail to exit the CNS despite elevated levels in the CSF [39, 41]. Furthermore, even when S100B does reach the bloodstream, it is rapidly cleared by the kidneys because of its low molecular weight (~ 21 kDa) and hydrophilic structure. Its plasma half-life is estimated at 1–2 h, which results in a steep decline in serum concentrations over time, especially when the rate of entry into the blood is reduced by impaired CSF-to-blood transport [42].

In contrast, NSE did not exhibit such divergence. Owing to its larger molecular weight (~ 78 kDa), NSE requires substantial structural BBB disruption to pass into the bloodstream [21]. Once such disruption occurs, NSE can move paracellularly into the serum rather than relying on glymphatic efflux [43]. Furthermore, because it originates from necrotic neurons and is passively released into the interstitial and CSF compartments, its availability for serum accumulation steadily increases with the extent of neuronal injury. NSE also has a longer plasma half-life of approximately 24 h, which allows for more sustained and detectable concentrations in the serum [9, 10, 44]. These factors together contribute to a synchronised increase in the serum and CSF concentrations of NSE, even under conditions of severe BBB disruption, without evidence of compartmental trapping.

Interestingly, 8 out of 44 patients with severe BBB disruption at H24 ultimately achieved good neurological outcomes. In 3 of these cases, severe disruption persisted through H72. However, we were unable to identify consistent clinical characteristics that could explain this apparent disconnect. These exceptions suggest that although severe BBB disruption is strongly associated with poor neurological outcomes, it is not invariably predictive. Further investigation is warranted to explore the underlying mechanisms and potential protective factors in such cases.

Our findings indicate that prognostic accuracy after cardiac arrest may not be determined by biomarker molecular weight or BBB integrity alone. Rather, biomarker-specific kinetics and compartmental behavior—such as active secretion, clearance dynamics, and serum–CSF dissociation—appear to play a substantial role. While our data underscore the potential value of accounting for both sampling time and BBB status, these findings are exploratory and hypothesis-generating. Prospective validation in larger cohorts is needed before such approaches can inform clinical prognostication.

### Limitations

This study has several limitations. First, it was a single-centre retrospective study with a relatively small sample size, which may limit the generalisability of our findings. Additionally, the study was not prospectively designed or powered as a dedicated diagnostic test accuracy trial. The performance metrics for biomarkers (e.g., AUC, sensitivity) were calculated post hoc as part of a mechanistic analysis and should be interpreted accordingly. A larger, multicentre, prospective study is needed to enhance the reliability and applicability of these results. Second, of the 174 OHCA survivors treated with TTM during the study period, 63 patients (36%) were excluded due to the absence of lumbar drainage catheter placement. This may have introduced selection bias and further limited generalisability. Third, a self-fulfilling prophecy bias cannot be excluded, as NSE and S100B results were available to treating physicians. However, WLST is not performed during TTM at our institution unless brain death is diagnosed. In this study, no patients underwent WLST during TTM, although some were declared dead based on circulatory or neurological criteria despite continued intensive support. Fourth, NSE measurements were prospectively collected from the beginning of the study, yielding a larger dataset (420 serum and 385 CSF samples) than S100B, which was introduced later (yielding 226 serum and 235 CSF samples). This discrepancy may have introduced statistical bias, although both biomarkers demonstrated prognostic value consistent with previous studies, suggesting limited impact on the primary findings. Fifth, CSF samples were not reanalyzed after dilution, and the assays used had restricted detection ranges. As a result, several patients with poor outcomes showed biomarker levels at or beyond the upper detection limits, potentially leading to underestimation of true concentrations and limiting further quantitative analysis. Sixth, the number of patients in each BBB disruption subgroup (intact, mild, moderate, and severe) was relatively small, reducing the statistical power of subgroup analyses. As such, caution is advised when interpreting biomarker performance across these subgroups. Finally, although lumbar catheterisation was initially performed under IRB-approved prospective protocols, it was later continued as part of routine clinical care with surrogate consent. This procedure is not standard practice globally, and CSF sampling was limited to patients meeting predefined safety criteria, which may introduce spectrum or selection bias. Future research into non-invasive assessments of BBB permeability—such as contrast-enhanced MRI—or complementary approaches is warranted [26]. Despite these limitations, this is the first study to evaluate biomarker molecular weight, BBB disruption, and kinetic compartmental behavior using paired serum and CSF data in OHCA survivors. These findings offer foundational insight for the refinement of biomarker-driven prognostication strategies in post-cardiac arrest care.

## Conclusions

In this study, we demonstrated that NSE and S100B were associated with neurological outcomes in OHCA survivors treated with TTM. However, their prognostic utility differed not by molecular weight or BBB dysfunction alone, but by biomarker-specific kinetics. NSE levels increased in parallel in CSF and serum as BBB disruption worsened and showed time-dependent improvements in predictive performance. In contrast, S100B remained elevated in CSF but declined in serum over time, with prognostic accuracy that did not consistently align with either BBB disruption severity or time post-ROSC.

These findings underscore the importance of interpreting biomarker dynamics beyond assumptions based solely on molecular size or barrier permeability. CSF-based biomarker analysis may serve as a valuable adjunct when serum markers yield inconclusive results. S100B appears most effective in early prognostication, while its diminishing serum levels may limit later interpretability. In contrast, NSE demonstrates consistently robust predictive performance across timepoints, supporting its integration into long-term multimodal prognostic models. This approach may assist clinicians in refining prognostication strategies by incorporating biomarker behavior across compartments and temporal phases. In particular, CSF concentrations of NSE and S100B showed superior discriminatory power over serum measurements, highlighting the value of compartment-specific biomarker assessment. These results call for additional prospective, multicenter, external validation studies for routine use of CSF biomarkers in postcardiac arrest prognosis.

## Electronic supplementary material

Supplementary Material 1

## Data Availability

No datasets were generated or analysed during the current study.

## Abbreviations

AUC: Area under the receiver operating characteristic curve
BBB: Blood–brain barrier
CCI: Charlson Comorbidity Index
CPC: Cerebral Performance Category
CNUH: Chungnam National University Hospital
CI: Confidence interval
CPR: Cardiopulmonary resuscitation
CSF: Cerebrospinal fluid
ECMO: Extracorporeal membrane oxygenation
FPR: False positive rate
HIBI: Hypoxic-ischaemic brain injury
IRB: Institutional Review Board
NSE: Neuron-specific enolase
OHCA: Out-of-hospital cardiac arrest
PCAC: Pittsburgh Cardiac Arrest Category
ROC: Receiver operating characteristic
ROSC: Return of spontaneous circulation
S100B: S100 calcium-binding protein B
TTM: Targeted temperature management
WLST: Withdrawal of life-sustaining therapy
