# Supplementary material for: Beyond blood-brain barrier disruption and molecular weight: compartmental kinetics of S100B and NSE for neurological prognostication after cardiac arrest
Source: Crit Care. 2025 Aug 4;29:342. doi: 10.1186/s13054-025-05572-8 (PMC12323214; doi:10.1186/s13054-025-05572-8)
Supplement: Supplementary file 1 — Supplementary Material 1 [file 13054_2025_5572_MOESM1_ESM.docx]

**Supplemental materials**

**Title:** **Beyond blood-brain barrier disruption and molecular weight: Compartmental kinetics of S100B and NSE for neurological prognostication after cardiac arrest**

**1. eAppendix 1. Table S1.** Temporal changes in albumin quotient according to neurological outcome

**2. eAppendix 2. Table S2.** Comparison of levels of NSE and S100B obtained from serum and CSF according to neurological outcome.

**3. eAppendix 3. Table S3.** Distribution of cause of death in patients with poor neurological outcome according to BBB disruption severity at 24 hours

**4. eAppendix 1. Figure S1.** Comparative analysis of neurological outcomes and BBB disruption post ROSC

**5. eAppendix 1. Figure S2.** Comparison of AUCs for predicting poor neurological outcomes 6 months after the ROSC

**1. Table S1.** **Temporal changes in albumin quotient according to neurological outcome**

| Day | Overall cohort (n = 111) | Good neurological outcome (n = 46) | Poor neurological outcome (n = 65) | *P-*value^a^ |
| --- | --- | --- | --- | --- |
| H0 | 0.0083 (0.0061–0.0132), 111^b^ | 0.0068 (0.0052–0.0095), 46^b^ | 0.0086 (0.0067–0.0164), 65^b^ | 0.004 |
| H24 | 0.0176 (0.0076–0.0500), 101^b^ | 0.0077 (0.0049–0.0155), 42^b^ | 0.0282 (0.0150–0.120), 59^b^ | <0.001 |
| H48 | 0.0140 (0.0069–0.0291), 94^b^ | 0.0069 (0.0059–0.0127), 41^b^ | 0.0233 (0.0133–0.0729), 53^b^ | <0.001 |
| H72 | 0.0149 (0.0068–0.0271), 93^b^ | 0.0075 (0.0055–0.0132), 40^b^ | 0.0228 (0.0147–0.0598), 46^b^ | <0.001 |

Continuous variables are presented as median (interquartile range)

^a^, P values are based on Mann-Whitney U test for continuous variables.

^b^, Number of patients included in the analysis

**Abbreviations**: H0 corresponds to immediately after return of spontaneous circulation, H24 corresponds to the 24 hours following H0 sampling,

H48 to the 24 hours following H24, and H72 to the 24 hours following H48

**Note**: “H0” represents the baseline sample collected at a median of 4.5 hours after ROSC.

**2. Table S2**. Comparison of levels of NSE and S100B obtained from serum and CSF according to neurological outcome.

| Time | Overall cohort | Good neurological outcome | Poor neurological outcome | *P-*value^a^ |
| --- | --- | --- | --- | --- |
| **Serum** | | | | |
| **Neuron-specific enolase, ng/mL** | | | | |
| H0 | 30.1 (20.8–43.1), 111^b^ | 24.6 (18.7–32.0), 46^b^ | 41.2 (28.2–76.9), 65^b^ | <0.001 |
| H24 | 32.1 (22.5–61.6), 105^b^ | 24.6 (19.5–34.5), 44^b^ | 54.0 (28.7–204.0), 61^b^ | <0.001 |
| H48 | 29.9 (19.3–92.0), 105^b^ | 21.6 (14.5–26.1), 46^b^ | 89.7 (28.0–225.0), 59^b^ | <0.001 |
| H72 | 31.1 (17.1–134.0), 99^b^ | 17.3 (12.9–25.0), 45^b^ | 91.3 (36.0–258.3), 54^b^ | <0.001 |
| **S100 calcium-binding protein B, ng/mL** | | | | |
| H0 | 0.47 (0.23–1.02), 59^b^ | 0.27 (0.16–0.42), 23^b^ | 0.80 (0.39–2.81), 36^b^ | <0.001 |
| H24 | 0.15 (0.08–1.18), 57^b^ | 0.09 (0.05–0.16), 22^b^ | 0.55 (0.14–2.39), 35^b^ | <0.001 |
| H48 | 0.14 (0.07–0.80), 57 ^b^ | 0.07 (0.05–0.13), 22^b^ | 0.38 (0.11–1.91), 35^b^ | <0.001 |
| H72 | 0.13 (0.06–0.51), 53 ^b^ | 0.06 (0.05–0.11), 22^b^ | 0.38 (0.09–1.36), 31^b^ | <0.001 |
| **Cerebrospinal fluid** |  |  |  |  |
| **Neuron-specific enolase, ng/mL** |  |  |  |  |
| H0 | 40.0 (19.4–130.0), 111^b^ | 19.9 (14.0–28.9), 46^b^ | 99.0 (41.4–204.5), 65^b^ | <0.001 |
| H24 | 291.8 (37.5–300.0), 98^b^ | 28.4 (13.6–65.1), 40^b^ | 300.0 (291.8–300.0), 58^b^ | <0.001 |
| H48 | 291.8 (25.3–300.0), 93^b^ | 23.7 (11.8–46.5), 40^b^ | 300.0 (292.9–300.0), 53^b^ | <0.001 |
| H72 | 291.8 (18.0–300.0), 83^b^ | 18.0 (12.9–42.6), 39^b^ | 300.0 (292.9–300.0), 44^b^ | <0.001 |
| **S100 calcium-binding protein B, ng/mL** | | | | |
| H0 | 3.66 (1.50–24.33), 68^b^ | 1.50 (0.90–2.02), 29^b^ | 15.70 (4.16–30.00), 39^b^ | <0.001 |
| H24 | 10.92 (1.46–30.00), 60^b^ | 1.30 (0.73–2.46), 22^b^ | 30.00 (30.00–30.00), 35^b^ | <0.001 |
| H48 | 7.97 (1.10–30.00), 57^b^ | 1.07 (0.67–1.51), 25^b^ | 30.00 (25.43–30.00), 32^b^ | <0.001 |
| H72 | 2.62 (0.68–30.00), 50^b^ | 0.71 (0.56–1.26), 24^b^ | 30.00 (9.61–30.00), 26^b^ | <0.001 |

Continuous variables are presented as median (interquartile range)

^a^, P values are based on Mann-Whitney U test for continuous variables.

^b^, Number of samples included in the analysis. The total number of patients included in the study was 111 (46 with good neurological outcome and 65 with poor neurological outcome).

**Abbreviations**: NSE, neuron-specific enolase; S100B, S100 calcium-binding protein B; CSF, cerebrospinal fluid; H0 corresponds to immediately after return of spontaneous circulation, H24 corresponds to the 24 hours following H0 sampling, H48 to the 24 hours following H24, and H72 to the 24 hours following H48

**Note**: “H0” represents the baseline sample collected at a median of 4.5 hours after ROSC.

**3. Table S3**. Distribution of cause of death in patients with poor neurological outcome (CPC 3–5) according to BBB disruption severity at 24 hours

| BBB disruption (at H24) | Total patients (n = 101) | Poor neurological outcome (CPC 3–5) | Neurological death (e.g., brain death, severe HIBI) | Extracerebral death (e.g., shock, MOF, sepsis) |
| --- | --- | --- | --- | --- |
| Intact | 23 (20.7%) | 4 (17.4%) | 1 | 3 |
| Mild | 14 (12.6%) | 6 (42.9%) | 2 | 2 |
| Moderate | 16 (14.4%) | 9 (56.3%) | 1 | 3 |
| Severe | 48 (43.2%) | 39 (81.3%) | 20 | 14 |

**Abbreviations**: H24 corresponds to 24 hours after return of spontaneous circulation; BBB, blood-brain barrier; CPC, Cerebral Performance Category;

HIBI, hypoxic ischemic brain injury; MOF, multi-organ failure

**Figure S1.** **Comparative analysis of neurological outcomes and BBB disruption post ROSC.**

(a) Comparison of neurological outcomes at 6 months post ROSC, stratified by BBB disruption. (b) Changes in the degree of BBB disruption over time following ROSC.

Blood and CSF biomarker and albumin samples were collected immediately after ROSC (H0) and subsequently at 24-h intervals over a 72-h period (H24, H48, and H72). The degree of BBB disruption according to the Q_A_ value: intact (Q_A_ < 0.007) or disrupted, which is further categorised as mild (Q_A_ 0.007–0.01), moderate (Q_A_ 0.01–0.02), or severe (Q_A_ > 0.02). ROSC, return of spontaneous circulation; BBB, blood-brain barrier

Note: “H0” represents the baseline sample collected at a median of 4.5 hours after ROSC.


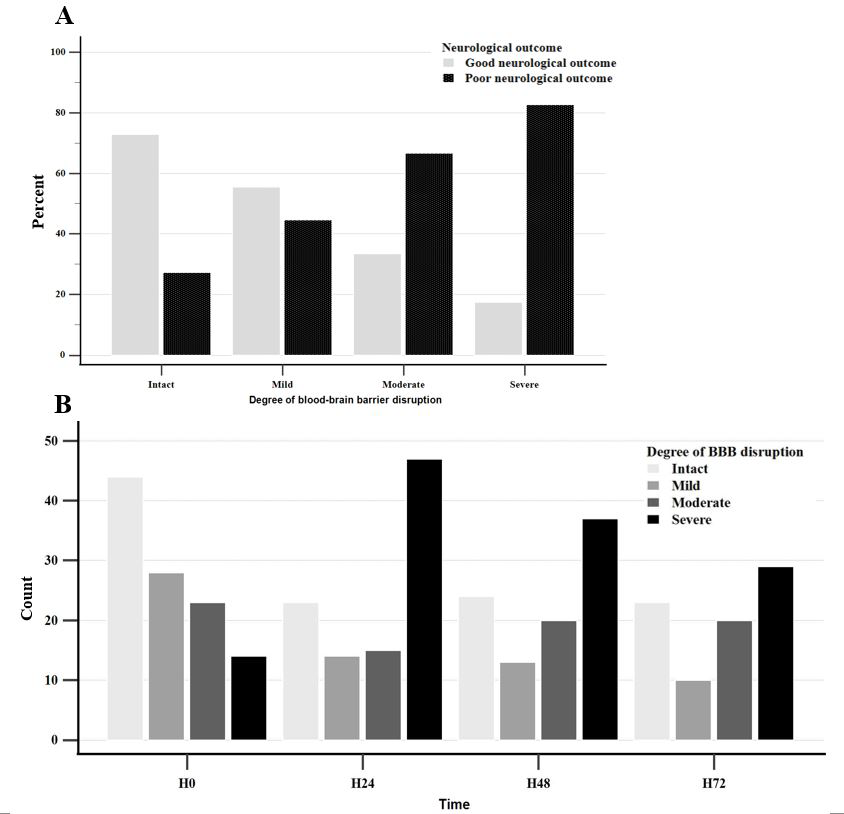


**Figure S2.** **Comparison of AUCs for predicting poor neurological outcomes 6 months after the ROSC.**

The comparison was based on serum concentrations of NSE and S100B measured at different timepoints. **(a**) ROC curves for serum NSE concentrations at H0, H24, H48, and H72. (b) ROC curves for serum S100B concentrations at the same timepoints. Blood biomarker samples were collected immediately after the ROSC (H0) and subsequently at 24-h intervals over a 72-h period (H24, H48, and H72).

^a^, Number of patients included in the analysis; ^b^, P*-*values are based on the DeLong test for AUC comparisons.

**Abbreviations:** AUC, area under the receiver operating characteristic curve; NSE, neuron-specific enolase; S100B, S100 calcium-binding protein B; CI, confidence interval

**
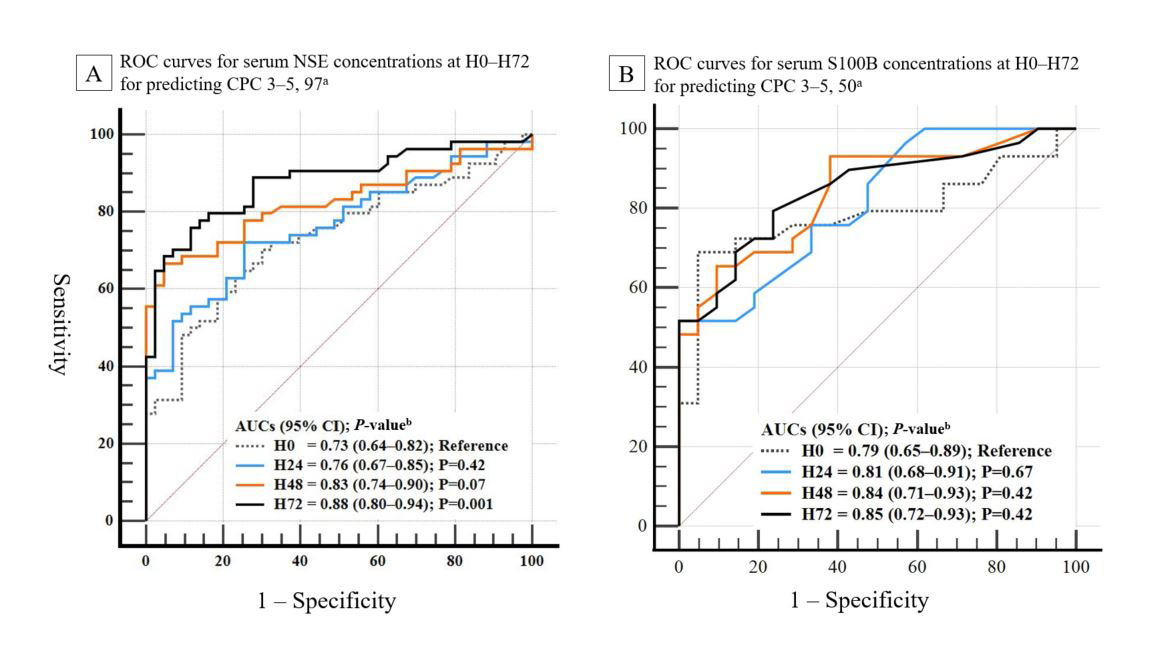
**
